# Fabrication and testing of polymer-based capacitive micromachined ultrasound transducers for medical imaging

**DOI:** 10.1038/s41378-018-0022-5

**Published:** 2018-08-27

**Authors:** Carlos D. Gerardo, Edmond Cretu, Robert Rohling

**Affiliations:** 1 Department of Electrical and Computer Engineering, University of British Columbia, 2332 Main Mall, Vancouver, BC Canada; 2 Department of Mechanical Engineering, University of British Columbia, 6250 Applied Science Ln, Vancouver, BC Canada

## Abstract

The ultrasonic transducer industry is dominated by piezoelectric materials. As an emerging alternative, capacitive micromachined ultrasound transducers (CMUTs) offer wider bandwidth, better integration with electronics, and ease of fabricating large arrays. CMUTs have a sealed cavity between a fixed electrode and a suspended metalized membrane. Manufacturing cost and sensitivity are limiting factors in current CMUTs that depend on the fabrication equipment and, especially, on the materials used. For widespread use of CMUTs, a much lower fabrication cost that uses inexpensive materials, which maintain or improve upon existing sensitivity, is needed. Herein, a new fabrication process is described for polymer-based CMUTs (polyCMUTs) using the photopolymer SU-8 and Omnicoat. The first ultrasound B-mode image of a wire phantom created with a 64-element linear array using synthetic aperture beamforming techniques is presented. A 12 *V*_AC_ signal superimposed on a 10 V_DC_ signal was used on the transmission side, and only a bias-tee, with no amplifiers, was used on the receiving side. The low operational voltage and high sensitivity of this device can be partially attributed to a pre-biasing condition on the membrane. By using a novel sacrificial layer combined with a top electrode embedded inside the membrane, we demonstrated that SU-8 can be used to manufacture CMUTs inexpensively. Moreover, the fabrication used relatively simple equipment, and the number of fabrication steps was reduced compared to traditional CMUT fabrication. This new fabrication process has the potential to increase the use of CMUTs in the ultrasound market, including the market for wearable transducers.

## Ultrasound imaging: plastic transducers

A procedure to fabricate capacitive micromachined ultrasound transducers (CMUTs) from plastic offers low cost and high performance. Ultrasound imaging typically relies on piezoelectric materials for transducers. However, their performance is inhibited by limited bandwidth, and they are challenging to implement in large arrays. Now, a team from University of British Columbia, Canada, led by Robert Rohling and Edmond Cretu reports a simplified and low-cost process for fabricating polymer-based CMUTs (polyCMUTs), which are attractive alternatives to their piezoelectric-based counterparts. Key to their process is encapsulating the electrode inside a membrane. The device works in a liquid medium, at low operating voltages, and at frequencies comparable to CMUTs fabricated from silicon nitride. PolyCMUTs could help to expand the use of ultrasound with flexible electronics.

## Introduction

Ultrasound imaging is the most widely used medical imaging modality in the world when considering the number of images created annually. The forecasted growth for the ultrasound market from US$4.6 billion in 2012 to almost US$7 billion by 2019 (ref. 1) has boosted research in medical ultrasound fields, especially with regards to transducer design.

The ultrasonic transducer industry is dominated by piezoelectric materials, with incremental development of the same basic transduction mechanism detailed almost a century ago. An appropriate acoustic impedance matching between the transducer and the medium is paramount in ultrasound imaging systems, as this impedance matching has a profound impact on the efficiency of the system. For current piezoelectric-based systems, there is a high acoustic impedance mismatch between piezoelectric crystals (~30 MRayl) and soft tissues (~1.5 MRayl). Typically, layers of high-density rubber, which act as an acoustic matching layer, are placed between the transducer and the medium. Although piezoelectric imaging transducers having fractional bandwidths of the order of 80% and higher are becoming gradually available, the majority of the imaging transducers exhibit limited bandwidths, typically in the range 30–50% (ref. 2).

Capacitive micromachined ultrasound transducers (CMUTs) have emerged as alternatives to piezoelectric imaging transducers^3^. A CMUT is essentially a parallel-plate capacitor with a fixed electrode at the bottom and a clamped metalized membrane suspended above a cavity. Ultrasound waves are generated when an AC signal, usually superimposed on a DC voltage, is applied between both electrodes; conversely, ultrasound waves can be detected by measuring the variation in capacitance of the device while a DC voltage is applied in the presence of incoming ultrasound waves.

CMUTs exhibit certain advantages over their piezoelectric counterparts, such as wider bandwidth, better integration with electronics, and ease of fabricating large arrays^4^.

The electromechanical coupling coefficient *k* characterizes the efficiency of an ultrasound transducer. For piezoelectric transducers the bandwidth is determined by *k* (ref. 5); while a high *k* is desirable for the useful vibration mode, a low *k* is often required for other competing modes, in order to suppress the spurious response caused by mode coupling^6^. This *k* value is not constant and depends on the shape and dimensions of the transducer^7^; other factors such as backing substrate, residual stress, and electrode coverage can also affect this coefficient^8^. In CMUTs, a high *k* leads to higher transducer sensitivity, improved bandwidth, and therefore improved image resolution^9^. Efficiency levels reported for CMUTs can be as high as 82% (ref. 10). The wide bandwidth for CMUTs is also the inherent result of the CMUT cell structure; the thin membrane clamped at the rim has a low mechanical impedance, which facilitates better acoustic matching to the medium^11^.

Conventional piezoelectric arrays are typically fabricated by mechanically dicing piezoelectric crystals and filling the kerfs with a polymer. Using this approach, the fabrication of ultrasonic arrays operating above 20 MHz is very challenging^12^. This contrasts with the wide range of operating frequencies of CMUTs, some of them reportedly reaching 60 MHz^13^.

In array operation, the parasitic capacitance of the interconnect between an element and its electronics is the limiting factor for the dynamic range and frequency bandwidth; therefore, it is preferred to have the electronics as close to the array elements as possible^14^. For piezoelectric two-dimensional (2D) arrays, a considerable sacrifice in the element area is required if an individual addressing is needed. The advantage of CMUTs in this respect is the fabrication of 2D arrays using through-wafer interconnections^14^ or trench-isolated interconnects^15^, where individual CMUT cells can be electrically accessed from the back side of the substrate. Another alternative is to use low-temperature processes that allow the fabrication of CMUTs directly above pre-existing components such as amplifiers and beamformers^16^.

Despite the advantages offered by CMUTs over piezoelectric transducers, they also have some important drawbacks. CMUT transducers suffer from acoustic crosstalk that contributes to image degradation caused by Stoneley waves at the membrane–water interface and Lamb waves propagating in the substrate^17^. Current CMUT designs have limited receiving sensitivity, which thereby reduces the signal-to-noise ratio and penetration depth into tissues^18^; this has been tested experimentally^19,20^, obtaining 10 dB less sensitivity for CMUT-based ultrasound probes compared to piezoelectric ones. Similar results regarding the penetration depth were obtained by Legros et al.^21^; nevertheless, the CMUTs outperformed piezoelectric probes in terms of the signal-to-noise ratio when phased-array steering was used; this was attributed to the larger acoustic radiation field experimentally measured from CMUTs.

A comprehensive and detailed description of the fabrication technologies for CMUTs is described by Ergury et al.^22^. From this review, surface micromachining and wafer-bonding technologies emerge as the most important alternatives. In surface micromachining, the cavity underneath the membrane is created by depositing or growing a sacrificial layer on the carrier substrate. After membrane deposition, the sacrificial layer is removed with an etchant that is specifically chosen to dissolve the sacrificial material via etch channels without damaging the membrane material^23^. In the wafer-bonding method, the membrane and the cavity are defined on separate wafers that are bonded together under vacuum conditions^24^. Given that etching channels are not required, the fabrication process is simplified, and a higher fill factor can be achieved.

Silicon nitride and polysilicon are the most popular materials for fabricating CMUT membranes, while chromium and aluminum are typically used to pattern electrodes on top of these membranes. These materials are chosen mainly for their mechanical properties, such that the membranes can be as thin as possible, in order to minimize the effective gap between the bottom and top (or hot) electrodes. By decreasing the effective gap between electrodes, the electric field can be increased, and the impedance matching to the electronics can be improved^22^. Once the desired operational frequency and the maximum biasing voltage have been identified, the CMUT membranes should be designed as thick as possible, given that their bandwidths linearly increase with thickness^25^.

Photopolymers are inexpensive, can be patterned using ultraviolet (UV) light, and have been used for the fabrication of microelectromechanical systems (MEMS) devices^26,27^. Their low density and high mechanical strength make them suitable for CMUTs because the impedance matching with the medium can be improved. Nonetheless, fabricating CMUTs using polymers is challenging because a thick membrane with a metal electrode on top is needed to reach the MHz region, contravening the required short gap between electrodes for low operational voltages and maximum sensitivity. Thus, despite some research in fabricating CMUTs using polymeric materials^28–30^, the operational voltages were in the order of hundreds of volts, making them less attractive to be used in biomedical ultrasound industries^31^. Moreover, the mentioned devices were reported to be only operating in air.

Despite the advantages of polymers for fabricating CMUTs, the main limitation for their applications is the large effective gap between electrodes and the requirement to be properly sealed for operating in fluids. Our solution for this problem was to embed the electrode inside the membrane to bring it closer to the bottom electrode, without sacrificing the overall thickness required to be in the MHz range.

Herein, we present a new fabrication process for CMUTs using polymeric materials and demonstrate for the first time the use of an operational CMUT array in a liquid medium. By encapsulating the electrode inside the membrane, instead of being on top, polymer-based CMUTs (polyCMUTs) were able to obtain low operational voltages and frequencies that were comparable to traditional CMUTs fabricated in silicon nitride or polysilicon.

The total estimated amortized manufacturing cost for the prototype polyCMUT array presented is below US$100. This is due to both the low cost of the materials used and the minimal manufacturing equipment required (mask aligner, metal evaporator, critical point dryer). Because the device is vacuum-sealed, the effective capacitance of the device is naturally increased by the atmospheric pressure as it deflects the membrane 27% of the total cavity height. Preliminary results indicate that these polyCMUTs can be pre-biased such that they can be operated even as passive devices (no external power) during reception and with low excitation voltages (10 V_DC_ + 12 V_AC_) during transmission.

The materials and solvents used during manufacturing have low toxicity, and the sealing layer covering the device is considered to be biocompatible. The maximum process temperature used was 150 °C, meaning that polyCMUTs can be potentially fabricated directly on top of substrates containing pre-existing components, such as beamformers and Tx/Rx switches. No acoustic matching layers were used. A novel sacrificial layer with high etching selectively was used, which can be spin or spray coated over rigid or flexible substrates, for wearable applications. The manufacturing process can be theoretically scaled up to roll-to-roll fabrication, decreasing the costs even further. This article is focused on the fabrication side of polyCMUTs that can be produced in a basic microfabrication facility.

## Materials and methods

### Material selection

In addition to improvements in the effective bandwidth, sensitivity, and radiated output pressure, a proper selection of fabrication materials can lead to improvements in the fabrication process and, in turn, lower the cost. We specifically selected the photopolymer SU-8 2000 series^32^ as a structural material for our applications, given its unique dielectric and thermal properties, as well as its low density, photopatternability, optical transparency, and mechanical flexibility.

A crucial component in the fabrication of the cavities required for CMUTs is the sacrificial layer, where a high selectivity of the etchant is required to properly dissolve the sacrificial layer without damaging the membranes.

Different materials have been used as sacrificial layers when working with SU-8. Song et al.^33^ used overbaked positive photoresists to withstand the attack of solvents present in SU-8 to form electroplating molds, and the sacrificial layer was later removed using plasma etching. Moser et al.^34^ altered the chemical composition of SU-8 to make it more sensitive to UV light, and a standard SU-8 composition was used as a sacrificial layer to create microchannels. Foulds et al.^35^ patterned polydimethylglutarimide (PMGI) to be used as a sacrificial layer, using a combination of positive photoresists and repetitive sequences of near UV and deep UV light exposures. Chiriaco et al.^36^ created microchannels by patterning PMGI without using deep UV light; the masking material for PMGI was removed in acetone, leaving the sacrificial layer prone to damage since acetone also attacks the PMGI. Finally, different metals have also been used as sacrificial layers^37,38^, where they are either evaporated or sputtered, requiring long deposition times and leaving rough surfaces. Metals also require extra fabrication steps to be patterned for use as sacrificial layers.

One of the main novelties of this work is the use of a well-known material in a novel way to create the CMUT cavities. Omnicoat^32^ has been widely used as a lift-off material to release structures from silicon wafers. It has an excellent selectivity during etching and enhances the adhesion of photoresists to different substrates.

Despite these properties, Omnicoat is not photosensitive and the typical thickness during spin coating ranges from 5 to 15 nm. Since a sacrificial layer in the order of hundreds of nanometers is needed, we evaporated 85% of the solvents present in Omnicoat. Thus, a much denser version of the same chemical was used for spin coating. Using this approach, we were able to increase the coating thickness from 15 to 300 nm in a single step at 1000 RPM. Multiple coatings could be stacked, or a higher percentage of the solvents could be evaporated if an even thicker sacrificial layer is desired. This might be useful in microfluidic applications where channels with different heights are typically used^39^. In our approach for CMUTs, the thickness of Omnicoat can be accurately controlled by spin coating, and it can be patterned at the same time as a masking material deposited on top. Omnicoat also has an excellent adhesion to different substrates, possesses excellent selectivity against solvents, and allows the substrates to be re-worked if necessary.

### Transducer fabrication

**Fig. 1:**
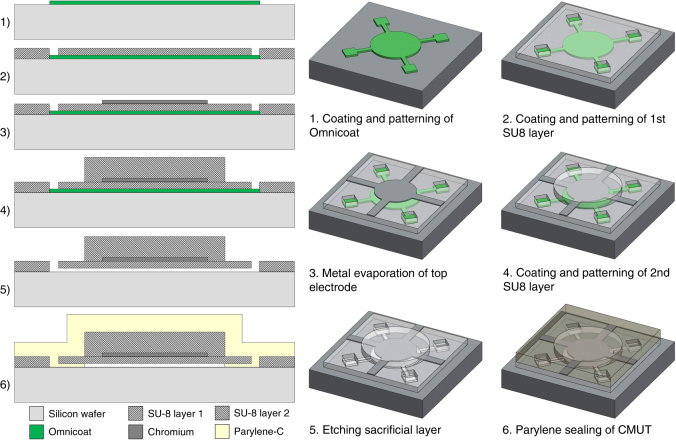
Overview of the six fabrication steps used to create polymer-based CMUTs

Fig. 1 depicts the fabrication steps for these devices. A maskless lithographic system SF-100 (St. Petersburg, USA) was used for UV exposure given its rapid prototyping capabilities; however, if needed, a standard mask aligner with photomasks can be used to replicate the results presented in this article.

The fabrication started with a clean and low electrical resistance silicon wafer that acts as the bottom electrode. We spin coated the concentrated version of Omnicoat (15% of its original volume) onto the wafer and baked it at 150 °C for 3 min to obtain a 320 nm sacrificial layer. A layer of positive photoresist S1813 (ref. 32) was then deposited and patterned using UV light to create a masking layer to selectively remove the Omnicoat underneath. The sample was then immersed in alkaline-based MF319 developer^32^ for 60 s, simultaneously etching the exposed areas of both S1813 and the Omnicoat; the etching was stopped by rinsing the sample in de-ionized water. The S1813 masking layer was then removed in acetone. Because of its excellent resistance to solvents, a patterned Omnicoat sacrificial layer was left intact without damage. A plasma etching step in O_2_ for 1 min is recommended at this time to completely eliminate all possible residues of Omnicoat left after etching and to increase adhesion of SU-8 to the silicon wafer. After plasma etching, the effective thickness of the sacrificial layer was 300 nm.

A layer of SU-8 2000.5 was spun at 2000 RPM onto the sample to obtain a thickness of 0.67 μm. The thickness facilitated conformal coating of the Omnicoat areas that became the cavities and etch channels of our device. A short pre-baking step at 95 °C was performed for 3 min prior UV exposure. The membranes, etching via holes, and clamping areas of the CMUTs were patterned in UV light, and the sample was post-exposure baked and developed. The SU-8 layer acts as a mechanical support for the top electrode and as dielectric material to avoid any short circuit since the dielectric strength of SU-8 reaches as much as 50% of that of SiO_2_ (ref. 40).

A layer of AZP 4110 photoresist was coated and patterned to act as a lift-off layer before evaporating on 570 nm of chromium. Chromium was selected because of its excellent adhesion properties to SU-8 films and low electrical resistivity^41^. A metal evaporation was preferred over sputtering because of its directional deposition, which simplifies the lift-off process. Once the electrodes are properly patterned, a second layer of SU-8 2002 was spin coated at 1000 RPM to obtain a thickness of 2.40 μm, thereby covering the previous stack of Omnicoat, SU-8, and chromium layers. After pre-baking, the sample was exposed to UV light to pattern the top part of the CMUT membranes, leaving open areas for the etching holes. The sample was cured at 150 °C for 5 min and then gradually cooled down to room temperature. This annealing step removes any possible cracks created and increases the Young’s modulus of the SU-8 films^42^.

The sample was then immersed in the developer MF319 (ref. 32). This etchant penetrates through the etch channels and gradually removes the Omnicoat material underneath the cavities. The circular cavities have a radius of 50 μm, and the etch channels are 5 μm wide and 15 μm long. After 3 h, the Omnicoat sacrificial layer was completely removed. The sample was then immersed in de-ionized water for 2 h to displace the MF319 developer trapped inside the cavities. Finally, the sample was immersed in isopropanol to replace the water.

A Tousimis (Rockville, MD, USA) critical point dryer was used to completely release the membranes, avoiding any stiction problems^43^. In order to make our membranes watertight, a 3.67 μm layer of Parylene-C was deposited in a low-pressure chamber, creating a sealed vacuum cavity inside the CMUT cell. In addition to providing excellent electrical insulation, Parylene-C is also biocompatible, optically transparent, has a low Young’s modulus, and has a coefficient of water absorption close to zero^44^. The dimensions of the fabricated CMUTs are specified in Table 1. The materials used allow for a visual inspection of the fabricated device, facilitating the identification of any possible defect.

**Table 1:** Parameters of fabricated polymer CMUTs

Component	Dimension
Membrane radius	50 μm
Sacrificial layer thickness	0.30 μm
Membrane SU-8 layer 1 thickness	0.67 μm
Top electrode thickness	0.57 μm
Membrane SU-8 layer 2 thickness	2.40 μm
Passivation layer thickness	3.67 μm

A slight modification in the process can lead to the fabrication of polyCMUTs on flexible substrates. Polyimide is a good candidate for this purpose given its elevated thermal stability, strong chemical resistance, and potential biocompatibility^45,46^; moreover, it has an excellent adhesion to metals and SU-8 films^41,47,48^. This polyimide film needs to be temporarily fixed to a rigid carrying substrate prior implementing the fabrication steps outlined in this article. This fabrication approach is still under investigation.

By using SU-8 and maskless lithography, a fully functional prototype was created in 17 h. This compares favorable to multi-user wafer fabrication services, such as MEMSCAP^49^ or MicraLyne^50^, where multiple designs are combined and processed with fixed fabrication protocols and long turnaround times. It is important to mention that the described fabrication process employs non-hazardous materials, that is, only organic solvents are used during manufacturing (acetone, isopropanol, SU-8 developer, and positive photoresist developer). The health risks associated with an accidental prolonged exposure to these materials do not go beyond drowsiness or minor skin irritation. This contrasts with the hazardous gas silane required to deposit silicon nitride and silicon dioxide in traditional CMUT fabrication^51^.

The final performance of the device is affected by several design parameters such as cavity height, membrane radius and thickness, electrode size, etc. The inherent resolution of the UV exposure system used allowed us to have release channels measuring 5 μm in width and 15 μm in length. Based on experimentation, a membrane radius of 50 μm gave us an acceptable fill factor while maintaining a low risk of stiction during releasing.

The CMUT cavity was designed to account for the natural deflection of the membrane caused by the atmospheric pressure after vacuum sealing. The remaining gap should be large enough to allow the membrane to vibrate during normal operation given the maximum allowed voltage.

The thickness of the first layer of SU-8 depends on the maximum operating voltage of the CMUT; it should be as thin as possible to maintain a small effective gap between electrodes while providing a good electrical insulation to prevent any voltage breakdown in case the membrane is brought in contact with the bottom electrode. A thermal annealing of this layer removes any possible microcracks and enhances the adherence of SU-8 to the substrate and to the subsequent chromium layer. A gradual cooling procedure is recommended as it reduces the risks of delamination.

Chromium was chosen as the top electrode material based on the excellent adhesion strength to SU-8 (ref. 41) and to the fact that it requires one single masking layer during lift-off. The metallization radius was set to 46% of the membrane radius in order to increase the bandwidth of the final device^25^. This metal layer should be as thin as possible to minimize its effects on the mechanical properties of the membrane. A 500 nm layer was sufficient to maintain a good electrical conductivity between electrodes. Having a layer too thin can result in resistive interconnection paths that, combined with the capacitance of the CMUT cells, would behave as a natural low-pass RC filter that degrades the driving signals during excitation.

The thickness of the second SU-8 layer is tailored to the desired operational frequency of the device. Since the effective gap between the top and bottom electrodes (and therefore the operational voltage) is dictated by the cavity height and thickness of the first SU-8 layer, this design allows the fabrication of low-frequency and high-frequency CMUTs with constant operational voltages. This contrasts with the design in traditional CMUTs where the operational voltage increases with membrane thickness. The thickness of this second SU-8 layer should also take into account the effects from the Parylene-C layer during vacuum sealing as it has similar mechanical properties as SU-8.

## Results and discussions

### Optical characterization

**Fig. 2:**
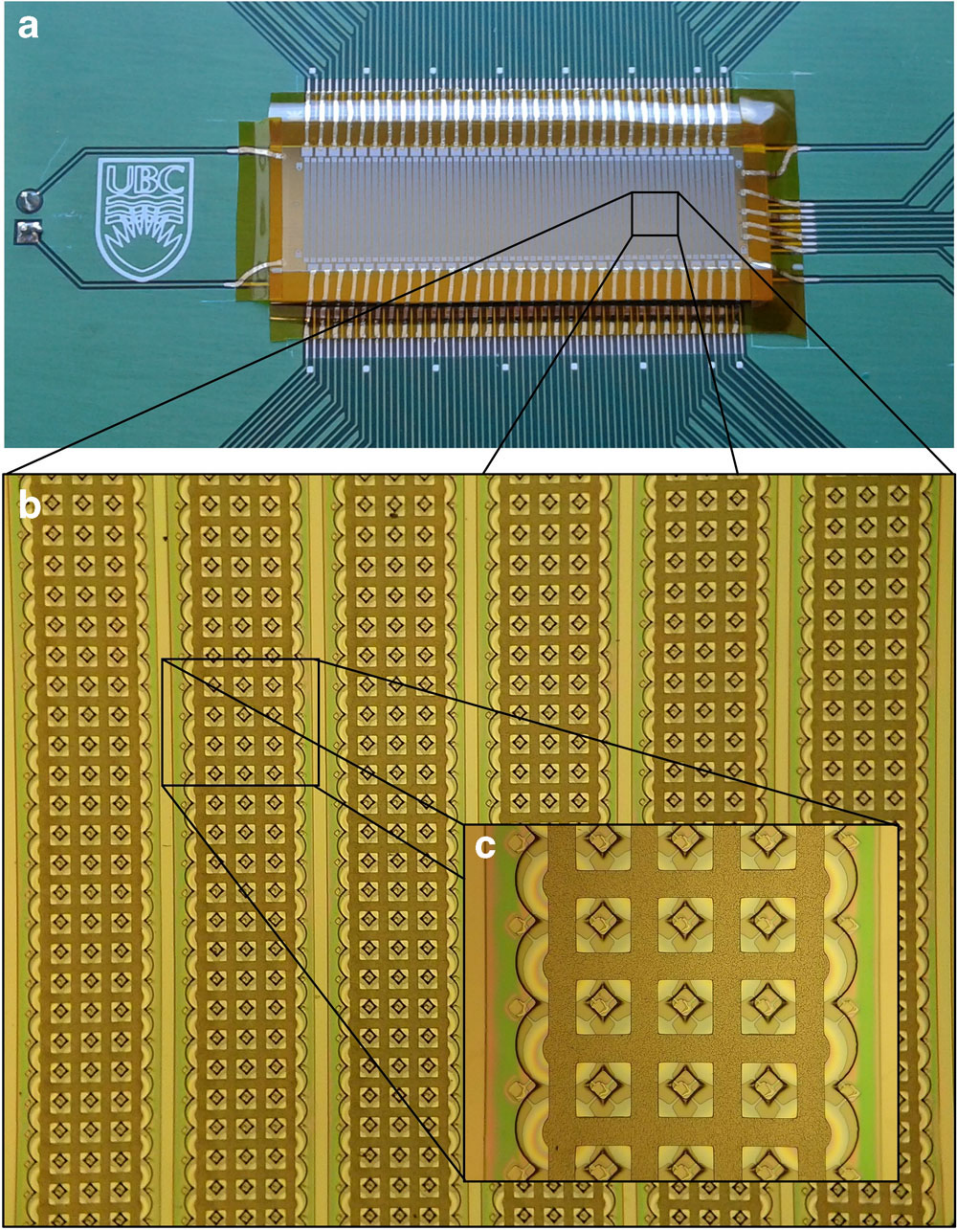
**a** Sixty-four-element CMUT linear array mounted on PCB. **b** Six CMUT elements separated by a pitch of 550 μm. **c** CMUT cells showing interconnected top electrodes and cavities underneath, the optical transparency of SU-8 allows a visual identification of any possible defect

**Fig. 3:**
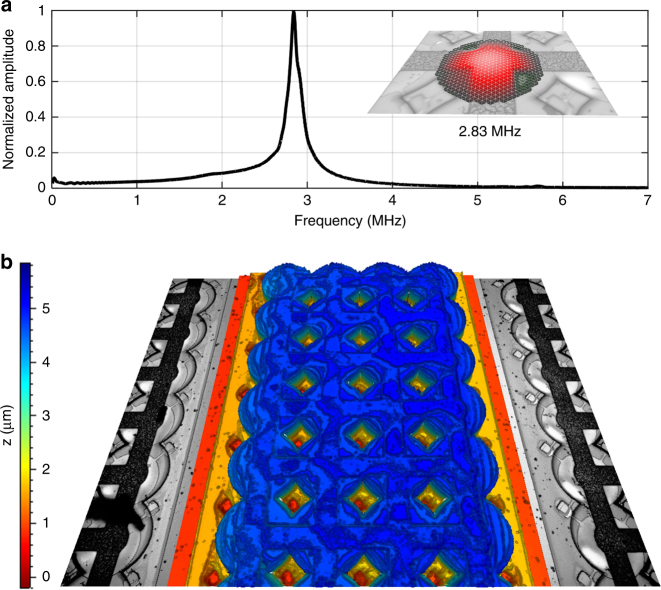
**a** In air characterization of polymer CMUTs showing the first vibration mode at 2.83 MHz. **b** Static topography of a CMUT element using white light interferometry

The final 64-element CMUT linear array was mounted and wired to a printed circuit board (PCB) interface circuit and is shown in Fig. 2. Each CMUT element contains 4 × 75 CMUT cells with a pitch of 550 μm between elements. The top electrodes on each element are interconnected in parallel. We characterized the frequency response of the sealed device in air using a laser Doppler vibrometer OFV-5000 (Polytec, Irvine, CA, USA). The frequency response of the system and the first vibration mode are shown in Fig. 3a, yielding a quality factor of 40 in air. The topography of the final device was characterized using a white light interferometer (Polytec, Irvine, CA, USA) and is shown in Fig. 3b with the thick SU-8 membranes of the CMUT cells displayed in blue.

### Validation of operation

**Fig. 4:**
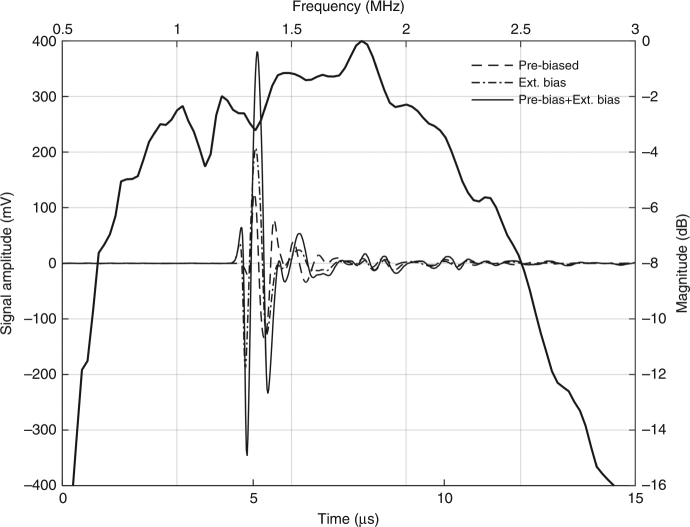
Acoustic response in mineral oil from polymer-based CMUT and FFT of received acoustic pulse showing a 101.6% fractional bandwidth

The CMUT array was immersed in a tank filled with mineral oil to avoid any electrical short circuits between wirings. A curved C5-2 broadband piezoelectric ultrasound probe (BK Ultrasound, Richmond, CA, USA) was used as an acoustical source, using an aperture of 64 elements to send a focused ultrasound beam to the polyCMUT array placed 50 mm beneath. A 50 V square wave was applied to the piezoelectric transducer to generate a single focused ultrasound wave that arrives perpendicular to the CMUT membranes. The measured pulse on the CMUT is shown in Fig. 4 under different biasing conditions. An external bias of 10 V was applied through a bias-tee. The pre-bias condition is explained in the next section.

The fast Fourier transform (FFT) of the measured pulse reveals that the 6 dB fractional bandwidth of this device is 101.6% around the center frequency of 1.525 MHz. This bandwidth for CMUTs is attributed to the relatively low fill factor of the design (59%), given that each individual cell pushes the fluid sideways, as well as in the normal direction. As a result, the hydrodynamic mass of the fluid for each cell increases and, therefore, reduces the center frequency and bandwidth^13^.

**Fig. 5:**
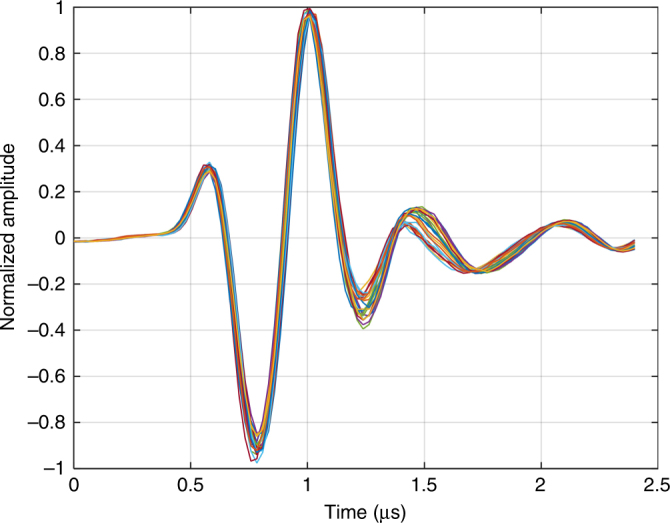
Ultrasound pulses generated by polymer CMUTs recorded every 30 min for a period of 12 h. Signals stayed within 95% of the maximum detected peak

An endurance test was performed in order to asses the durability of the fabricated device. A 2 MHz sine wave superimposed on a 10 V_DC_ was used to excite the polyCMUT linear array using a pulse repetition frequency of 5 kHz. The piezoelectric probe acted as receiver and recorded the detected pulses every 30 min for a period of 12 h of continuous operation, the results are shown in Fig. 5. Even though the polymer CMUT array underwent over 200 million excitation cycles, the amplitude of the signals stayed within 95% of the maximum detected peak. Further endurance tests involving high temperatures and elevated voltages are still under way.

### Pre-biasing

Charge trapping in CMUTs occurs when electrical charges are trapped in insulating layers in a CMUT membrane. This phenomenon was initially considered as a negative effect in CMUTs because it shifts the resonant frequency of the membrane over time^52^. However, Park et al.^53^ demonstrated that these electrical charges in CMUTs can be introduced in a well-controlled manner and used to operate a CMUT without any DC bias applied. The main benefit of having electrical charges trapped in the CMUTs is that they act as a built-in bias voltage, which suggests that only a small external DC voltage (compared to the required voltage if no trapped charges were present) is required to bring the membrane closer to the bottom electrode, that is, the effective gap between electrodes is reduced, thereby increasing its capacitance. As this effective capacitance is increased, a small perturbation to the membrane deflection caused by external acoustic sources will result in a large variation in the capacitance.

Direct detection, without amplification of ultrasound waves coming from the same piezoelectric transducer described in the previous section, was possible owing to this pre-biasing condition. Even though no external voltage is applied between the CMUT terminals, a 260 mV_PP_ echo signal was detected, as shown in Fig. 4.

The first SU-8 layer described in the fabrication steps has a dual purpose. First, it serves as an insulation layer between the top and bottom electrode, preventing a short circuit in case the membrane is brought in contact with the top electrode. Second, the SU-8 layer acts as a novel charge trapping layer. For the first time, SU-8 was used to retain electrical charges, acting as a built-in bias voltage.

As determined by experimentation, electrical charges are trapped in the CMUT membrane and create an intrinsic (built-in) bias voltage. This contributes to the natural deflection of the CMUT membrane and, therefore, lowers the external DC bias voltage required for actuation and reception. Following a similar procedure as used by Park et al.^53^, the CMUTs were “charged” by applying a DC voltage of 100 V between the top and bottom electrodes for 5 min. During this time, the membrane collapsed against the bottom electrode, generating an electric field strength of 1.49 MV/cm. After removing the bias voltage, the CMUT was connected to an impedance analyzer to obtain a *C*–*V* curve. The symmetry point of this *C*–*V* curve revealed that an intrinsic (built-in) voltage of 8.9 V was present. From this, an average trapped charge of 7.25 × 10^−8^ C/cm^2^ was calculated. Larger intrinsic bias voltage could be experimentally obtained by charging the CMUTs for longer periods of time, for example, charging the CMUT for 210 min leads to a 30 V pre-bias condition.

The SU-8 membrane is naturally deflected towards the bottom electrode as a result of the atmospheric pressure. This serves to bring the top and bottom electrodes closer to each other, thereby increasing the capacitance. As the membrane becomes increasingly thin, this effect is increased^54^.

**Fig. 6:**
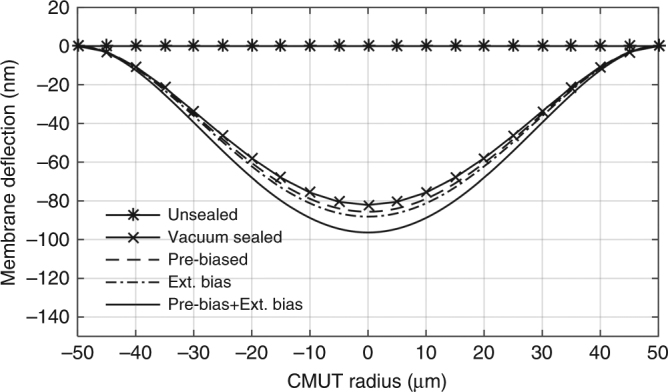
Contribution of different factors towards the total CMUT membrane deflection

Figure 6 shows the contribution of different effects towards the total deflection of the membrane. Atmospheric pressure is a major influence. Naturally developed by sealing the cavity in a vacuum chamber, atmospheric pressure deflects the membrane 82 nm (27% of cavity height) towards the bottom electrode. The pre-biasing condition (built-in bias voltage) increases the deflection by 1.5%. Lastly, the contribution of the external bias voltage increases the total membrane deflection to a maximum of 32.1% of the cavity height.

By embedding the electrode inside the membrane, we were able to obtain a pull-in voltage of 65 V, which contrasts with the 220 V required when the metal electrode was placed directly above the CMUT membrane as in traditional CMUT designs.

### B-mode imaging

**Fig. 7:**
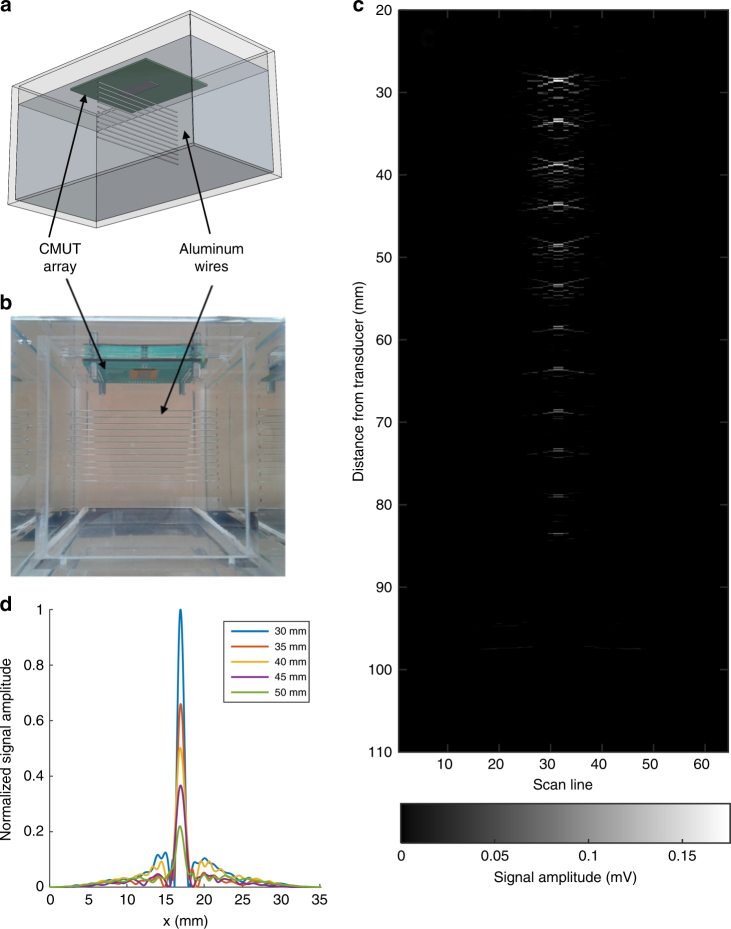
**a** Diagram of the wire phantom used; 12 aluminum wires spaced by 5  mm. **b** Photograph of the actual wire phantom immersed in mineral oil. **c** B-mode image of a wire phantom using a linear array of polymer-based CMUTs using a display dynamic range of 30 dB. **d** Assessment of the lateral resolution, the FWHM for the first 5 wires down to a depth of 50 mm was <1.5 mm

The feasibility of using these polyCMUTs for the creation of B-mode images was assessed. An array of 12 aluminum wires with a diameter of 600 μm equally spaced by 5 mm was used as a target in a tank filled with mineral oil as shown in Fig. 7a, b. The linear CMUT array was placed directly above the wires and excited by a 12 V_PP_ square pulse that was oscillated at 2 MHz and superimposed on a 10 V_DC_ bias using a bias-tee. This external bias voltage was selected in order to avoid pull-in in case the CMUT was charged for a long period of time.

Only one CMUT element was used during transmit, thereby creating a quasi-cylindrical wavefront. The echoes created by the aluminum wires were captured by all other elements. For receiving, a 10 V_DC_ bias was applied to the CMUT using a bias-tee. The echoes were measured by a digital oscilloscope, and averaged over 256 times to reduce electrical noise.

The received signals were later processed in MatLab (Natick, MA, USA) using synthetic aperture beamforming techniques^55^. A simple Hanning window was applied as apodization and no time-gain compensation processing was used. A B-mode image generated using polyCMUTs is shown in Fig. 7c. Despite the unamplified measurement scheme, all of the 12 wires in the phantom can be identified to a depth of 85 mm. The signal profiles of the first 5 wires of the phantom were taken in order to assess the lateral resolution of the polyCMUT array as shown in Fig. 7d, the full-width at half-maximum revealed a resolution of <1.5 mm.

## Conclusions

This work provides a proof of concept that polymeric materials can be used to fabricate CMUTs for biomedical imaging. Having the top electrode embedded inside the membrane led to low operational voltages comparable to traditional CMUTs fabricated in polysilicon or silicon nitride. A 64-element CMUT linear array was successfully operated and characterized, leading to the first demonstration of a B-mode image created with polyCMUTs.

An advantage of using Omnicoat as sacrificial material is that the deposition method is simple and controllable. The thickness of this layer can be reduced to a few tens of nanometers if desired, leading to potentially increased sensitivity and even lower operational voltages. This is the first demonstration where a condensed version of Omnicoat is selectively patterned and used as sacrificial layer for SU-8.

The materials used were selected in such a way as to manufacture CMUTs inexpensively, minimize the fabrication steps, and obtain similar dimensions comparable to traditional CMUTs^22^. This work highlights the benefits of using a simple fabrication process with inexpensive materials coupled to basic microfabrication equipment. Although SU-8 photopolymer was used for this study, any other polymer or soft material can in principle be used for the fabrication of CMUTs. The important factor is to maintain a small effective gap between the electrodes of CMUTs for low operational voltages and high sensitivity.

A full acoustic characterization for the presented polyCMUTs is yet to be done. Performance tests for these devices are under way to create a pre-biasing mathematical model.

This technology has the potential to be extended to flexible substrates intended for conformal and wearable health monitoring systems^56,57^. This contrasts with traditional fabrication approaches that use a combination of semiconductors and metals as structural materials. Although flexible CMUT arrays have been derived from rigid substrates^58,59^, problems have been reported with electrical interconnections of individual elements getting cracked.

An advantage of this design is that the low-temperature process (max 150 °C) can be seamlessly integrated with CMOS-compatible processes for closer integration with electronics. SU-8 can also be easily coupled with other materials, such as polydimethylsiloxane or polymethylmethacrylate, for microfluidics applications^60,61^.

A variation of the proposed fabrication methodology is to create the same version of polyCMUTs using wafer-bonding technologies for SU-8, as reported by Joseph et al.^62^. The fabrication methodology can also potentially be extended to roll-to-roll technology^63^, where CMUT cavities and membranes are defined on separate flexible substrates and then bonded together under a vacuum environment. This could decrease production times and costs even further, with the possibility of fabricating ultra-low-cost ultrasound transducers.
